# Age estimation of burnt human remains through DNA methylation analysis

**DOI:** 10.1007/s00414-024-03320-1

**Published:** 2024-09-13

**Authors:** Pierangela Grignani, Barbara Bertoglio, Maria Cristina Monti, Riccardo Cuoghi Costantini, Ugo Ricci, Martina Onofri, Paolo Fattorini, Carlo Previderè

**Affiliations:** 1 Dipartimento di Sanità Pubblica, Medicina Sperimentale e Forense, Università di Pavia, Pavia, Italy; 2 Dipartimento di Scienze Biomediche, Metaboliche e Neuroscienze, Università di Modena e Reggio Emilia, Modena, Italy; 3 AOU Careggi SOD Diagnostica Genetica Equipe Genetica Forense, Firenze, Italy; 4 Dipartimento di Medicina e Chirurgia, Azienda Ospedaliera S. Maria, Università di Perugia, Terni, Italy; 5 Dipartimento Clinico di Scienze mediche, chirurgiche e della salute, Università di Trieste, Trieste, Italy

**Keywords:** DNA methylation, Age estimation, Burnt remains, Single base extension (SBE)

## Abstract

The identification of human fire victims is a challenging task in forensic medicine. The heat-induced alterations of biological tissues can make the conventional anthropological analyses difficult. Even if the DNA profile of the victim is achieved, it is possible that no match can be found in a forensic DNA database, thus hindering positive identification. In such cases, any information useful to nail down a possible identity should be collected, such as DNA methylation analysis which could provide useful investigative leads. In the present study, five age-related epigenetic markers (ELOVL2, FHL2, KLF14, C1orf132, and TRIM59) were initially analysed in blood samples of 72 living Italian individuals of known age, using a Single Base Extension (SBE) assay. An age prediction model was built by multiple linear regression including all the markers (Mean Absolute Error, MAE: 3.15 years). This model was tested on 29 blood samples collected during autopsies from burnt human remains, already identified through DNA analysis, providing a MAE of 6.92 years. The model allowed a correct prediction in 79.3% of the cases (95% prediction interval), while six cases were associated with inaccurate predictions (min-max prediction error: 9.8–37.3 years). Among the different sample variables considered to explain these results, only the DNA degradation index was a relevant factor affecting the reliability of the predictions. In conclusion, the SBE typing of blood from burnt remains proved to be a reliable tool to estimate chronological age of most of the samples, also in consideration of its cost-effectiveness and the availability of CE sequencers in every forensic genetics laboratory.

**Supplementary Information:**

The online version contains supplementary material available at 10.1007/s00414-024-03320-1.

## Introduction

Identification of burnt human remains can be a challenging task, depending on the severity of the thermal alteration. In suicidal or accidental burnings, car accidents, mass casualties (for examples airline accidents), the impact of fire on bodies can vary from the most extreme situation where the biological tissues are completely destroyed, preventing any attempt of identification, to different modulations of fire induced modifications on bodies as described in Glassman and Crow [1] and Pope et al. [2]. Depending on the fire environments (e.g., residential structure fires, vehicle fires, confined space fires, and outdoor space fires), characteristic sequential heat-related changes to the layered tissue can be observed, resulting in a variability of fire alterations among the anatomical regions in the different situations.

There are many factors affecting the heat-induced alterations on bodies; among these the most important ones are the fire/flame temperature, the duration of exposure, the exposure/position of the body and its proximity to fire, and the presence of clothing or protective objects or items accelerating or delaying the burning. These factors may influence the sequential events, resulting in an altered burn pattern or a differentiated burning of the body [2].

In such cases, the reconstruction of a biological profile of the victim can be pivotal to achieve relevant data linking to a possible identity of the body. Sex, age-at-death, ancestry, and stature, as well as further information useful for identification purposes, such as personal descriptors/identifiers, dental profile, and medical features (e.g. prosthesis, antemortem fractures, and congenital or traumatic deformities or abnormalities), are generally collected during post-mortem examinations.

A further aid to the reconstruction of the biological profile of a burnt body can arise from the most recent advances in DNA typing of external visible characteristics (EVCs) markers described in the recent review by Kayser et al. [3]. Eye, hair, and skin colour prediction based on DNA have been deeply studied and predictive tools have been validated [4], while, more recently, DNA methylation pattern analysis seem to be a very promising biomarker for age estimation [3].

The combination of EVC and epigenetic DNA markers can be very useful in forensic investigations on unrecognizable burnt bodies as it can provide investigative leads especially when the corresponding autosomal STR profile does not find a match in a forensic DNA database.

In many autopsies of burnt human remains, after removing the external carbonized tissue, it is common to find blood either in liquid form or “coagulated” by heat. It can be collected from body cavities, blood vessels, or after heart incision and the extracted DNA can be processed to achieve autosomal STR profiles useful for individual identification and/or to characterise the methylation state of age-related CpG target sites [5] for chronological age estimation.

Many papers have already investigated age-related epigenetic markers on blood collected from living donors [6–9] and different technologies [10–12] and aged prediction models [13–16] have been proposed. The methylation state of CpG sites in five genes (namely ELOVL2, FHL2, KLF14, C1orf132, and TRIM59) has been found to strictly correlate to the chronological age of individuals in fresh blood [6, 7]. The accuracy of the age prediction varied in different populations and using different methylation analysis techniques with a deviation from chronological age of approximately 3.17–5.35 years [6, 7, 15, 17].

What is still largely unknown is the effect of post-mortem alterations on DNA methylation patterns especially for aged stains and severely damaged bodies as it is expected to vary depending on the state of preservation of the human specimens [18, 19].

To this aim, different studies have investigated the topic in cadaveric blood stored at -20 °C and collected from individuals with a post-mortem interval within 10 days, targeting the same or different age-correlated markers and using various technologies [20–22]; the final age prediction models resulted in a deviation from chronological age ranging from 5.23 to 7.60 years.

In this paper, the age prediction ability of the CpG sites ELOVL2, FHL2, KLF14, C1orf132, and TRIM59 was first tested in a sample set of fresh blood collected from living Italian individuals. Then, in order to understand the impact of heat-induced alterations on the age estimation, the methylation state of the same five age-related CpG sites was investigated in blood samples recovered from differently burnt human remains, according to the classifications by Glassman and Crow and Pope et al. [1, 2]. The methodological approach selected was the semi-quantitative single base extension (SBE) technique, also known as minisequencing or SNaPshot sequencing, for its ability to detect multiple CpG sites simultaneously, for the cost-effectiveness of the technique compared to the more expensive microarray or NGS approaches and, in particular, for the widespread availability of automatic DNA sequencers based on capillary electrophoresis (CE) in all forensic laboratories. This is because age prediction of forensic biological samples based on DNA methylation cannot be considered a routine test as, in most cases, it is requested in single casework analysis (for example to provide investigative leads useful for the identification of burnt human remains). For its occasional frequency, the test can be more easily and cheaply set up in a forensic lab by using DNA CE sequencers instead of other more expensive technological approaches such as EpiTyper or NGS.

## Materials and methods

### Ethic statement

The present project study was approved by the Ethic Committee of the University of Trieste (Comitato Etico di Ateneo, number 135, October 24^th^, 2023) and all samples were immediately anonymized.

### Blood samples

#### Living individuals

Peripheral blood samples were collected from 72 healthy Italian individuals from North-Eastern Italy, among which 34 and 38 were males and females, respectively. The age range spanned from 18 to 85 years with a mean age and standard deviation (SD) of 49.3 +/- 18.9 years. The blood samples were immediately stored at -20 °C until use. All individuals signed an informed consent allowing the use of their samples for research purposes.

#### Burnt human remains

Blood was collected from 29 burnt human remains during autopsy at the Pavia and Trieste Institutes of Legal Medicine, between 2010 and 2023. The blood tissue was either liquid or solid (thermally coagulated by heat) and was found inside body cavities (mainly in the torso region) or after dissection of internal organs (mainly heart) or blood vessels; in the most compromised bodies, blood was so dehydrated to look like a dark red sandy powder. Genetic identification of the burnt human remains has been achieved, in general, within one month after the finding of the bodies through autosomal STR comparison with the genotypes of the victim’s relatives or with the genotypes recovered from personal items used by the victim. The age of the identified subjects ranged from 24 to 75 years with a median age and SD of 49.9 +/- 15.3 years. The victims were 21 males and 8 females. The post-mortem interval (PMI), that is the time elapsed from death to the finding of the remains, was in most cases < 24 h; in two cases it was 48 and 72 h, respectively, while in one single case it was unknown. The time from the presumptive death to the collection of the blood samples during autopsies (TDC) was available for 28 out of 29 samples and ranged from 1 to 97 days (median = 5, IQR = 5).

### Body preservation/thermal damage classification

Thermal changes to the body were evaluated according to the classification systems proposed by Glassman and Crow [1] and Pope et al. [2]. Five levels (from level 1 to 5) and six stages (from stage 1 to 6, each one divided in early [A] and advanced [B] changes) describe increasing levels of thermal changes, from superficial burning injuries to extensive damage with fragmented bones and little or no tissue still present. While the Crow-Glassman Scale (CGS) outlines the overall burning conditions of a body, the Pope and colleagues’ classification system provides descriptions for each anatomic region (i.e., head, torso, upper and lower limbs). Since most of the blood samples were collected from the torso region, the Pope’s system was applied according to the description of this region. To this purpose, pictures taken during the external examination of the body were examined.

### DNA extraction

DNA was extracted from a 500 µl aliquot of blood samples collected from living individuals using the conventional phenol/chloroform/isoamylic alcohol extraction method. If liquid blood was recovered from the burnt remains, a 250 µl aliquot was extracted, according to the protocol of the QIAmp DNA Mini kit (Qiagen); in case of solid or sandy blood, the samples were weighted and 25–30 mg were then extracted following the *DNA purification from tissue protocol* described in the user’s manual of the same kit. DNA extracted from the burnt remains was resuspended in 200 µl of AE buffer. Blank controls were always included.

### DNA quantification

#### Fluorimetric DNA determination

The DNA recovered from blood samples of the living individuals was quantified using the QuantiFluor^®^ ONE dsDNA System kit (Promega) on a Quantus™ Fluorometer (Promega), according to the manufacturer’s protocol. Briefly, 1 µl of the extracted DNA was mixed with 199 µl of QuantiFluor^®^ ONE dsDNA Dye and, after a 5-minute incubation protected from light, the samples’ concentrations were measured.

#### Molecular DNA quantification (qPCR)

The DNA quantification of the blood samples recovered from the burnt human remains was performed in duplicate experiments on the 7500 Fast Real-Time PCR System (Thermo Fisher Scientific), using the PowerQuant™ System kit (Promega) and following the manufacturer’s instructions. DNA quantification values were normalised according to the short amplification probe (Auto). The ratio between the Auto/Deg autosomal probes values (that is the degradation index, DI) was calculated for each sample. Negative (no DNA) and positive controls (commercial DNA samples with known concentrations) were always included. Data were analyzed with the Applied Biosystems 7500 Analysis Software 2.3 (ThermoFisher Scientific).

### DNA methylation assay

Bisulfite conversion of the DNA samples and the following steps of multiplex PCR amplification of the five CpG sites ELOVL2, FHL2, KLF14, C1orf132, and TRIM59, Exo-SAP purifications and single base extension (SBA) were exactly the same as described in the paper by Onofri and coworkers [23]. Briefly, 100–400 ng of DNA were treated with sodium bisulfite according to the EZ DNA Methylation-Direct^TM^ Kit (ZymoResearch, Irvine, CA, USA) protocol; 1–4 µl (approximately 40 ng) of the corresponding converted DNA were added to the multiplex PCR for the amplification of the five selected CpG sites. Duplicate PCR amplifications were set up for each converted DNA. Accordingly, the primer sequences for PCR amplification and the probes extended in the SNaPshot assay were as described in [7, 17], while the corresponding working concentrations were the same reported in [23]. One microliter of the primer extended products was mixed with 10 µl of HiDi formamide and 0.2 µl of LIZ 120 internal size standard (Thermofisher), denatured at 95 °C for 2 min, and separated by capillary electrophoresis on a SeqStudio Genetic Analyzer Instrument (Thermofisher). The methylation state of the five CpG islands was scored using the GeneMapper^®^ Software v 6. The analytical threshold for data interpretation was 30 rfu.

### Calculation of the methylation levels

The ratio of the methylated and unmethylated bases at each CpG site was calculated according to [17] for each replicate PCR.

DNA methylation levels were calculated as the mean of the two replicates for each sample of the living and burnt human remains samples.

### Statistical analyses

Statistical analyses were carried out using RStudio software (RStudio 2023.12.1 + 402 “Ocean Storm” Release (RStudio Team (2015). RStudio: Integrated Development for R. RStudio, Inc., Boston, www.rstudio.com/).

The relation between age-at-death and DNA methylation levels was evaluated using the Pearson correlation test and was further investigated by univariate linear regression. A blood age estimation model was subsequently developed using multiple linear regression including all the CpG sites as predictors. Results were illustrated in the Forest plot using the *ggstatsplot* package [24]. Model performance was evaluated through repeated k-fold cross-validation.

An exploratory sub-model was developed adding sex as an additional independent variable. Goodness-of-fit and accuracy of the models were assessed by assessing several quantitative measures, such as the R^2^ coefficient, mean absolute error (MAE), root mean square error (RMSE), Akaike Information Coefficient (AIC), and Bayesian Information Coefficient (BIC).

The model was subsequently used to estimate the age-at-death (95% prediction interval) of the blood samples collected from the 29 burnt human remains and the accuracy of the prediction was assessed by computing the MAE value. Association analyses were performed using logit/univariate linear regression in order to identify any relation between the correctness of the prediction/prediction error (i.e., the difference between estimated and chronological age) and sample variables (i.e., thermal damage score, fire environments, time from death to sample collection or TDC, and degradation index).

Statistical significance was assessed when the p value was lower than 0.05.

## Results

### Living donors

A training set of 72 living individuals from 18 to 85 years old was selected to develop the age prediction blood model based on the analysis of the five CpG sites ELOVL2, FHL2, KLF14, C1orf132, and TRIM59. DNA was extracted from blood samples and quantified by a fluorimetric assay. 400 ng DNA were bisulfite converted and duplicate PCR amplifications of the selected CpG islands were set up. The methylation state of the selected markers was assessed through Single Base Extension assays using the SNaPshot kit (Online Resource 1). For each sample, the electrophoretic separations of the extended probes in duplicate experiments were performed, to test the repeatability of the assays.

In the living individuals’ blood samples, all the five CpG sites showed a significant correlation between DNA methylation levels and age. Specifically, a positive correlation was observed for ELOVL2 (*r* = 0.9470), FHL2 (*r* = 0.9432), KLF14 (*r* = 0.7062), and TRIM59 (*r* = 0.8849), while C1orf132 was negatively correlated with age (*r*= -0.8945). The observed relationships were confirmed by univariate linear regression. A graphical representation of the relationships and a summary of the linear regression statistics for each site are reported in Figure S2 (Online Resource 2).

**Fig. 1:**
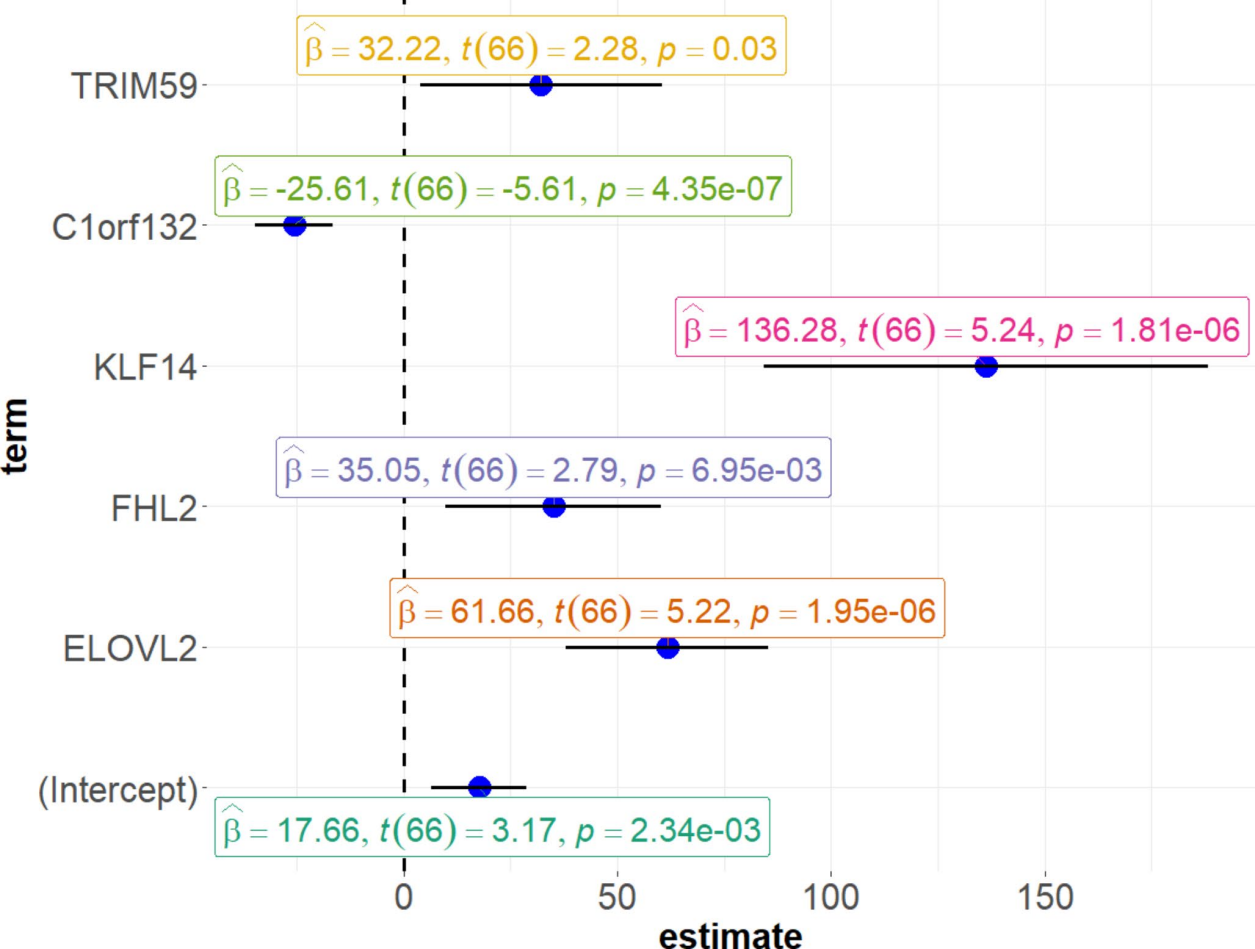
Forest plot representing the estimated regression model coefficients (β) and 95% confidence intervals with the corresponding t-statistic (t) and associated *p*-value (p) explaining the statistical significance of each model term

**Fig. 2:**
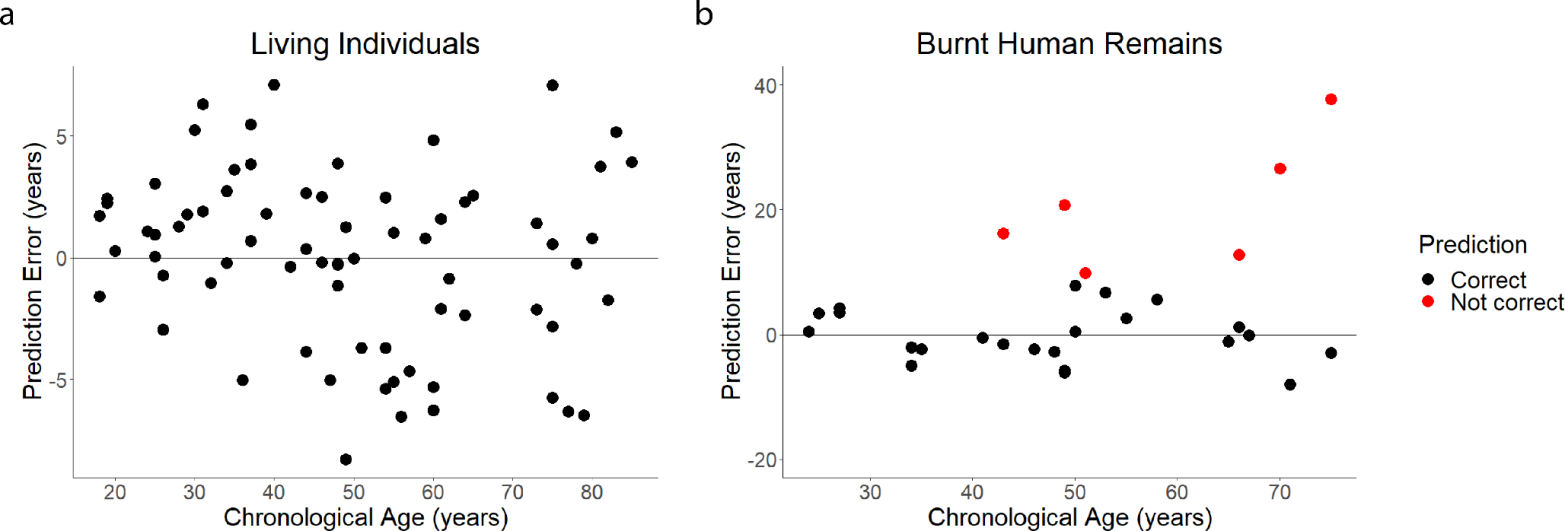
Prediction error versus chronological age for the living **(a)** and burnt human remains **(b).** Correct and not correct predictions are shown in black and red, respectively

All the sites were therefore included in the computation of a multivariate linear regression. Results showed a significant p-value associated with the model (F stat = 357.4, p-value = 2.30e-46), a significant correlation with age for the five predictors, and a coefficient of determination (R^2^) equal to 0.964 (adjusted R^2^ = 0.962). The summary statistics of the model are reported in Fig. 1 and Table S1 (Online Resource 3). The pure testing of the whole dataset resulted in a Mean Absolute Error (MAE) of 2.84 and a Root Mean Square Error (RMSE) of 3.55 for the blood samples collected from living individuals. Plotting the prediction error *versus* chronological age an increase of the error with age was detected (Fig. 2a). Splitting the living sample into four age groups (i.e., 18–34, 35–49, 50–64, > 65), the youngest group showed a MAE value of 1.98, while older categories had values equal to 2.88, 3.28, and 3.38, respectively.

An exploratory sub-model was developed adding sex as a further independent variable. Since no significant relationship was observed between the added variable and age and no substantial differences were detected in the model statistics (Tables S2-S3, Online Resource 3), sex was excluded as a predictor. The final blood age estimation model included therefore all five CpG sites and resulted in the following prediction formula:

$$\begin{gathered}\:Age = {\mathrm{17.66}} + \left( {{\mathrm{61.66}} \times \:DNA{m_{ELOVL2}}} \right) + \left( {{\mathrm{35.05}} \times \:DNA{m_{FHL2}}} \right) \hfill \\\,\,\,\,\,\,\,\,\,\,\,\,\,\,\,\,\,\,\,\,\,\,\,\,\,\,\,\, + \left( {{\mathrm{136.28}} \times \:DNA{m_{KLF14}}} \right) + \left( {{\mathrm{25.61}} \times \:DNA{m_{C1orf132}}} \right) \hfill \\\,\,\,\,\,\,\,\,\,\,\,\,\,\,\,\,\,\,\,\,\,\,\,\,\,\,\,\, + \left( {{\mathrm{32.22}} \times \:DNA{m_{TRIM59}}} \right) \hfill \\ \end{gathered}$$

Where $\:{DNAm}_{(.)}$ is the DNA methylation level of the specified CpG site.

Model validation was carried out through repeated k-fold cross-validation. In particular, the sample was randomly cleaved into k subsets (k = 5), k-1 subsets were used for training the model and the remaining subset for assessing model performance. The process was repeated three times. The analysis yielded the following performance parameters: R^2^ = 0.962, MAE = 3.15, and RMSE = 3.88. Table S4 (Online Resource 3) reported a summary of the accuracy of the model and model validation.

### Burnt remains

A test set of 29 blood samples collected during autopsy from burnt human remains was selected in order to check the age prediction model developed on the living donors. The carbonization state of each burnt individual was determined on the basis of two different classification systems; the first, proposed by Glassman and Crow [1] with a score (from 1 to 5), reported increasing levels of thermal modifications of the body tissues; the second, by Pope et al. [2], provided scores on the basis of the descriptions of the heat-related modifications of each anatomic region. For most of the human remains, blood was found in internal organs (such as the heart) and collected still in the liquid state; in a few cases, blood was found in a “solid” state inside blood vessels as for a heat-caused protein denaturation. In one single case of extremely burnt remains showing the highest scores for the Glassman and Pope systems, blood was collected as a sandy red powder. In most cases death had been caused by suicide or car accidents. With regards to the fire environment where the heat-related event occurred, approximately two-thirds were inside a vehicle, followed by 27% in a residential environment (i.e., inside a building) and in one single case it was outdoors, in an open space. A positive identification of the victims was achieved for all the burnt remains through STR-based DNA profiling and direct comparisons with personal items or through kinship analysis.

Human DNA was quantified through a qPCR assay using the commercial kit Promega PowerQuant™ System.

In most cases, the 400 ng of DNA needed for the bisulfite conversion could be obtained from the blood samples, while in few cases, only 100–150 ng of DNA could be achieved. A quality parameter of the integrity of the extracted DNA was determined through the calculation of the Degradation Index (DI), which is the ratio between two autosomal probes with different lengths. According to the interpretation guidelines of the quantification kit, DNA samples with DI < 2 are to be considered not degraded, while DI values ≥ 2 highlight increasing degradation patterns. The DNA samples recovered from the burnt remains showed a mean DI value and standard deviation of 1.88$\:\pm\:$1.26 (median value 1.46, min and max values 0.82 and 6.30) pointing out a substantial integrity of the DNA structure (at least, according to the fragmentation of the DNA).

Similarly, duplicate PCR amplifications and the following steps were carried out also for the blood samples from the burnt human remains (Online Resource 1).

All the demographic details and molecular data of the samples, together with the carbonization scores, are reported in Table 1.

**Table 1:** Details and molecular data of the burnt samples. **PMI**: post-mortem interval; **TDC**: time (days) from death to sample collection; **Glassman score**: thermal damage classification according to Glassman and Crow [1]; **Pope score**: thermal damage classification according to Pope et al. [2]; **fire environment** (O: outdoor space fire, R: residential structure fire, V: vehicle fire); **blood feature**: feature of the blood sample (L: liquid, SC: solid-coagulated, S: sandy); **DI**: DNA degradation index

					Carbonization				
Sample	**Age**	**Sex**	**PMI**	**TDC**	**Glassman** **score**	**Pope** **score**	**Fire environment**	**Blood** **feature**	**DI**
1	25	M	< 24 h	3	2	1 A	V	L	1.43
2	24	F	< 24 h	3	2	1 A	V	L	1.17
3	49	M	48 h	8	5	5B	R	SC	1.57
4	66	M	< 24 h	3	3	3 A	R	L	1.14
5	75	F	< 24 h	8	5	6 A	V	S	1.47
6	53	M	< 24 h	5	4	3B	V	L	1.56
7	55	M	< 24 h	5	1	1 A	V	L	1.60
8	46	M	< 24 h	97	4	5 A	V	SC	1.14
9	65	M	NA	NA	2	1 A	O	L	1.46
10	43	M	< 24 h	2	4	3 A	V	L	3.73
11	66	M	< 24 h	5	3	2B	R	L	6.30
12	51	M	< 24 h	7	1	1 A	R	L	1.04
13	41	M	< 24 h	1	4	5B	V	L	0.85
14	50	M	< 24 h	3	2	1 A	V	L	1.14
15	58	M	< 24 h	3	2	2 A	R	L	1.00
16	70	F	< 24 h	13	1	1 A	R	L	3.34
17	67	F	< 24 h	4	3	3 A	R	L	1.52
18	43	F	< 24 h	9	2	2B	V	L	3.89
19	49	F	< 24 h	9	3	3 A	V	L	3.43
20	27	F	< 24 h	9	3	2B	V	L	1.52
21	49	M	< 24 h	5	3	3 A	V	L	3.57
22	50	M	< 24 h	2	4	4B	V	L	1.75
23	48	M	< 24 h	4	4	4B	V	L	0.82
24	27	M	< 24 h	7	2	2 A	V	L	0.91
25	34	F	< 24 h	4	4	4B	V	L	1.18
26	35	M	< 24 h	4	3	2 A	V	L	1.34
27	34	M	< 24 h	4	3	2B	V	L	2.29
28	71	M	< 24 h	6	2	2 A	V	L	1.03
29	75	M	72 h	10	5	5B	R	SC	1.21

The blood age estimation model was then used to predict the chronological age from the blood samples of the 29 burnt human remains. Since the chronological age was known for all the identified individuals, the prediction accuracy was evaluated. Prediction error was calculated by subtracting the chronological from the predicted age providing values between 0.18 and 37.71 years (MAE = 6.92). Figure 2b depicts the prediction error *versus* chronological age for each individual. By computing a 95% prediction interval, age was correctly estimated in 79.3% of cases (23 out of 29). Among the six cases associated with an inaccurate prediction, one sample (number 12) had a slightly higher prediction range than the chronological age (only 0.9 years from the lower value, prediction error = 9.8 years). The remaining five cases showed instead larger prediction errors, between 12.8 and 37.7 years.

In order to determine which factors affect the model accuracy in our dataset of burnt remains, four sample variables were considered (i.e., thermal damage scores, fire environments, time from death to sample collection or TDC, and degradation index) and related to the correctness of the prediction and prediction error through logit/univariate linear regression. Since low variability was observed for the blood samples’ features and PMI, these variables were not included in the analyses.

Only the degradation index (DI) showed a significant association (*p* = 0.018 and *p* = 0.048 for the correctness of prediction and prediction error, respectively; see Table S5, Online Resource 3). In particular, a cut-off value of 2 was detected by a classification and regression tree (CART) analysis. Blood samples with DI values equal/lower than 2 showed an incorrect prediction in 9% of the cases (2 cases not correctly predicted out of 22 cases with DI ≤ 2), while the percentage increased for blood samples with greater DI values (57%, 4 cases incorrectly predicted out of 7 cases with DI > 2). No association was observed between DI values, thermal damage scores, fire environments, and TDC.

In conclusion, the analyses showed that there was no evidence that body preservation, fire environments, and TDC influence model performance, whereas the model was able to correctly predict age mainly for DI values ≤ 2 for the PowerQuant™ System kit.

## Discussion

The study of burnt human remains is a challenging task in forensic medicine for many reasons. Depending on the fire environment, different modulations of fire-induced modifications can be observed on the bodies. The first purpose is to determine the identity of the victim which can be pursued by comparing post-mortem and antemortem data (e.g., odontological, anthropological, medico-legal, and/or genetic data), or, in the cases of no information about the identity of the victim, by creating a biological profile to target suspects of identity.

In defining a biological profile the age-at-death is one of the most important issues. In forensic anthropology, practical guides have been provided for ageing the dead, suggesting the best approaches according to age ranges and body preservation states [25, 26]. However, when dealing with burnt human remains, the burning rate and thermal changes to the skeleton may affect the applicability of the methods proposed and the accuracy of the results [25, 27]. When dealing with adults, further questions should be considered. In fact, regardless of the state of preservation of the body, ageing adults is more difficult than ageing subadults: as skeletal maturation is reached, methods rely on the physiological degeneration of skeletal and dental structures, resulting in wider error ranges, and therefore large age ranges, with the increase of age. A further question concerns the impossibility to distinguish adults over 60 with the commonly used (traditional) age estimation methods, making these methods less helpful in the elderly [26, 28, 29].

The evaluation of the DNA methylation pattern at specific CpG sites can support conventional anthropological and medico-legal investigations in order to provide a reliable range of age prediction of the burnt human remains.

To this aim, a set of the five most age-related CpG markers (ELOVL2, FHL2, KLF14, C1orf132, and TRIM59) was studied in a training set of blood samples collected from 72 living Italian individuals spanning from 18 to 85 years, using the Single Base Extension (SBE) methodology. All markers showed individually a significant correlation with the chronological age and, specifically, a positive and negative correlation for the CpG sites ELOVL2, FHL2, KLF14, TRIM59 and marker C1orf132, respectively. Then, a statistical age prediction model was built through a multiple linear regression equation including the methylation state of all the 5 CpG sites resulting in a MAE value of 3.15 years. Other authors have typed the same set of methylation markers in blood samples from living individuals in different population samples using different methods [17, 20, 23] and showed very similar mean prediction errors. In addition, the same trend of lower accuracy in the age prediction for older age ranges [20] was highlighted also in our sample set of living individuals with MAE values from 1.98 for the younger set of individuals to 3.38 for the older one.

The age prediction model developed in this study was used to test its ability to correctly estimate the age of burnt human remains, starting from blood collected inside internal organs or blood vessels during autopsies. Twenty-nine blood samples were recovered from burnt remains showing different levels of thermal changes to the body, evaluated according to the classification systems proposed by Glassman and Crow [1] and Pope et al. [2].

Our data showed that 23 out of 29 DNA samples from burnt remains (79.3%) can be included in the 95% prediction interval with a MAE value for the sample set of burnt remains of 6.92 years. Similar mean prediction errors were reported in another paper [20] where other post-mortem blood samples collected during autopsies were typed for the same set of markers and characterised with the same SBE methods. The remaining 6 samples showed conversely relevant differences between the predicted age, as calculated by the methylation patterns, and the chronological age, with absolute errors ranging from 9.8 to 37.7 years, thus highlighting a general overestimation of the predicted over the chronological age. In order to rule out technical errors in the bisulfite conversion, amplification and primer extension steps, and considering that SBE is a semi-quantitative method [11], these 6 blood samples were re-extracted and re-submitted to the same typing steps described previously confirming the methylation ratios for the selected markers. Many could be the factors affecting the accuracy of the age estimation for these samples, among these different fire-induced changes in the blood samples affecting the primary structure of the DNA and leading to a reduction of the amount of DNA and to a possible alteration of the methylation ratio for the selected CpG markers. However, degradation does not seem to be a critical issue for most of the DNA samples extracted from burnt remains, as stated by the mean and median values of the DNA degradation index which revealed a substantial integrity of the DNA structure (at least in the range of the two probes of the PowerQuant quantification kit) and only a slight decrement of the molecular weight of the template. Nevertheless, DNA fragmentation (that is the breaking of phosphodiester bonds) is only one of the mechanisms of post-mortem DNA damages among which nucleobases deamination is one of the most studied, especially in ancient [30, 31] and forensic samples [32, 33]. Concerning the present study, cytosine and 5-methylcytosine deamination, generating U and T respectively, could alter the detection of the real methylation ratio at one or more specific CpG sites. In addition, even if some anamnestic data on the deceased were available, no relevant information about lifestyle habits (for example food and alcohol consumption, stress, smoking, exposure to air pollution) and general health conditions were available for most of the burnt remains. In fact, it is well known that these risk factors [34–36] or other unknown genetic backgrounds can be associated with accelerated epigenetic ageing, thus leading to significant differences between the epigenetic and the chronological age. It is remarkable that two of the samples whose age was not correctly predicted were two sisters who perished in a fatal food truck fire. The corresponding chronological age of the sisters was 43 and 49 while the predicted age from the methylation patterns resulted 59 and 70, respectively. Given that the fire conditions, the degradation index (3.89 and 3.43 for the two samples), the TDC (which was 9 days), and the analytical lab steps were the same for the two sisters, these significant deviations from chronological ages could be explained speculating the same effect of the fire conditions and PMI on DNA methylation patterns and/or a shared epigenetic background among sisters; this condition could have produced the same methylation modifications leading to an epigenetic age acceleration of the DNA in both full siblings.

On the other hand, the case of the “correct” age prediction of the burnt remains belonging to a male driver who perished in a track fire is also noteworthy. While the burnt remains (Glassman and Pope scores 4 and 5 A, respectively) were recovered immediately (PMI < 24 h), the remains were stored in the cold room of the morgue for 97 days at + 2 °C, until the putative relatives of the deceased could be found for the genetic identification. Nevertheless, the blood sample was of good quality and a not degraded DNA was recovered, showing a low degradation index value (DI 1.14) which allowed the achievement of an autosomal STR profile useful for genetic identification and of the DNA methylation pattern for the selected CpG markers providing in the end a rather good prediction of the correct chronological age (error: 2.38 years).

## Conclusions

The recent achievements in epigenetic pattern analysis nowadays allow us to make reliable age predictions from different human fluids and tissues of forensic interest. While, at the beginning, most of the statistical models were being developed starting from “fresh” biological samples such as blood, saliva, and semen collected from living individuals [6, 7, 15, 17, 20, 37–40], the most recent applications also include blood [20–22], bones [40–42], teeth [18, 41, 42], and costal cartilage from deceased subjects and bloodstains [43–46]. This is the challenging aspect of forensic epigenetics as the modifications of the DNA methylation patterns in post-mortem tissues are largely unknown but need to be studied and evaluated in order to rely on the age prediction calculated from the epigenetic statistical models. Post-mortem influencing factors include temperature, PMI, how long and where the body was stored before autopsy, and the impact of transformative cadaveric processes. Recent studies however report that DNA methylation seems to be stable up to 48–72 h in post-mortem samples [47, 48], even if these studies are only based on animal models. Other papers have described the results obtained on bloodstains [43, 44] and post-mortem tissues [40–42, 45, 46] supporting the possibility of a reliable age estimation of human cadaveric tissues.

To this aim, we have developed a statistical age prediction model based on five CpG markers analysed in blood collected from 72 living individuals testing it for accuracy and reliability in the age prediction of blood samples collected from burnt human remains.

Finally, we correctly predicted the age in about 80% (79.3%) of the burnt human remains with a MAE for the sample set of burnt remains of 6.92 years, which can be considered a good result for a potential application in forensic casework, such as DVI scenario, to distinguish family members with a genetic relationship detected among severely burnt victims (e.g., parent-child).

Nevertheless, the remaining 20% of cases resulted in inaccurate age prediction with even significant differences from the chronological age of the deceased (from 9.8 to 37.7 years). Overall, these results confirmed that, at least according to our data, the most relevant factor affecting the reliability of the correct age prediction is the integrity of the DNA [47] as assessed by the DNA degradation index value. In fact, only 9% of the burnt remains whose age was inaccurately predicted showed DI values equal/lower than 2, which is the threshold above which a sample starts to be considered degraded. Other factors were investigated without finding any correlation in our sample set of burnt remains, among which the carbonization scores according to forensic anthropological classifications, the fire environment, and the time lapse between death and collection of the samples during autopsy (TDC). One of the limits of the present study is represented by the small number of burnt human remains which might influence the statistical significance of the results obtained. Other factors probably need to be investigated in order to understand and estimate the impact of post-mortem changes on the DNA methylation patterns thus reducing misleading age evaluations.

The Single Base Extension method selected in this study to determine the DNA methylation patterns proved to be reliable, cost-effective and perfectly fitting a non-routine use of the assay for age estimation in most of the forensic laboratories where DNA CE sequencers represent the basic equipment.

## Electronic supplementary material

Below is the link to the electronic supplementary material.

Supplementary Material 1

Supplementary Material 2

Supplementary Material 3

## Data Availability

The datasets generated during the current study are available from the corresponding author upon reasonable request.
