# Supplementary material for: Age estimation of burnt human remains through DNA methylation analysis
Source: Int J Legal Med. 2024 Sep 13;139(1):175–85. doi: 10.1007/s00414-024-03320-1 (PMC11732892; doi:10.1007/s00414-024-03320-1)

AGE ESTIMATION OF BURNT HUMAN REMAINS THROUGH DNA METHYLATION ANALYSIS

Pierangela Grignani[1], Barbara Bertoglio[1*], Maria Cristina Monti[1], Riccardo Cuoghi Costantini[2], Ugo Ricci[3], Martina Onofri[4], Paolo Fattorini[5], Carlo Previderè[1]

[1] Dipartimento di Sanità Pubblica, Medicina Sperimentale e Forense, Università di Pavia, Pavia, Italy
[2] Dipartimento di Scienze Biomediche, Metaboliche e Neuroscienze, Università di Modena e Reggio Emilia, Italy
[3] AOU Careggi SOD Diagnostica Genetica Equipe Genetica Forense, Firenze, Italy
[4] Dipartimento di Medicina e Chirurgia, Azienda Ospedaliera S. Maria, Università di Perugia, Terni, Italy
[5] Dipartimento Clinico di Scienze mediche, chirurgiche e della salute, Università di Trieste, Trieste, Italy

[*]Corresponding author: Barbara Bertoglio, Laboratorio di Genetica Forense, Dipartimento di Sanità Pubblica, Medicina Sperimentale e Forense, Università di Pavia, via Forlanini, 12, 27100 PAVIA, Italy, email: barbara.bertoglio@unipv.it

Fig S1 Primer extension reactions (electropherograms) defyning the methylation ratio of two individuals analysed in the present study. A) Blood sample from a 65 years old living individual. B) Blood sample from burnt human remains belonging to a 66 years old individual. The methylated/unmethylated bases for each of the five selected CpG markers are shown below the corresponding peaks.

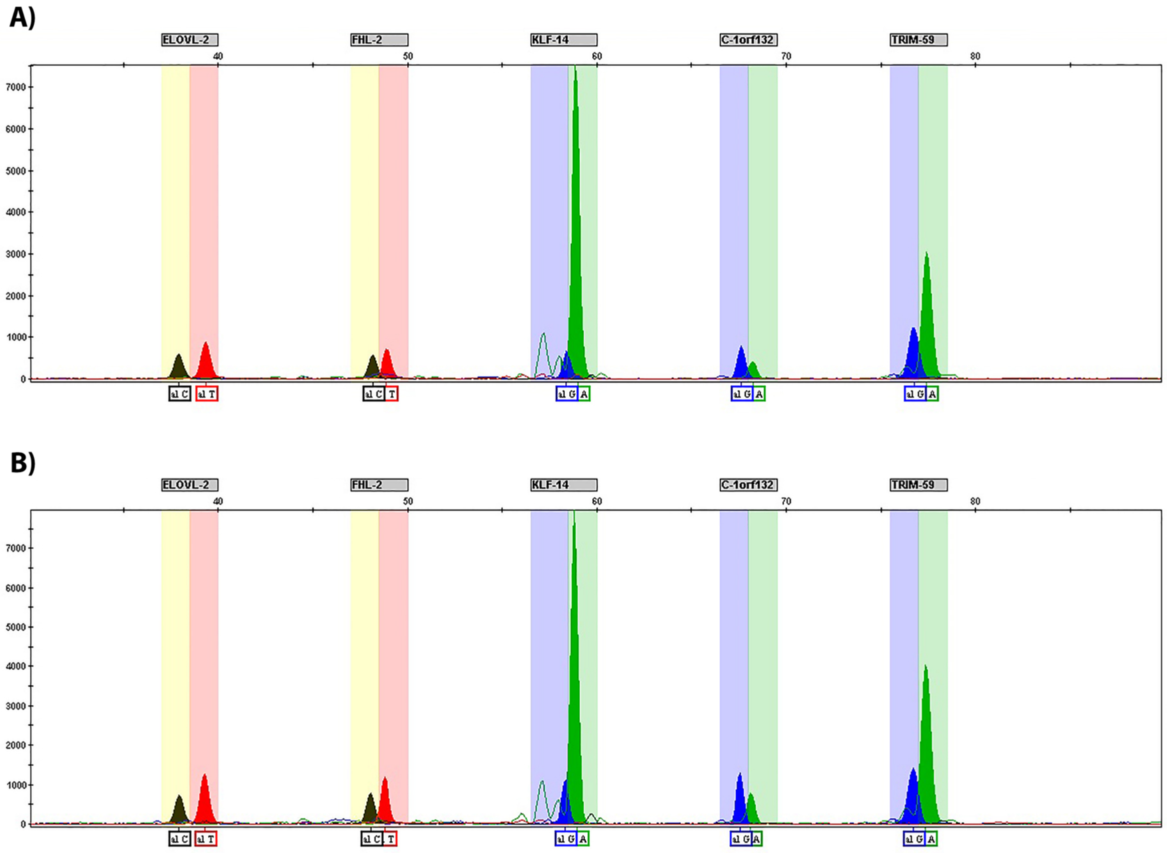

Supplement: Supplementary file 1 — Supplementary Material 1 [file 414_2024_3320_MOESM1_ESM.pdf]
