# Supplementary material for: Age estimation of burnt human remains through DNA methylation analysis
Source: Int J Legal Med. 2024 Sep 13;139(1):175–85. doi: 10.1007/s00414-024-03320-1 (PMC11732892; doi:10.1007/s00414-024-03320-1)
Supplement: Supplementary file 2 — Supplementary Material 2 [file 414_2024_3320_MOESM2_ESM.pdf]

AGE ESTIMATION OF BURNT HUMAN REMAINS THROUGH DNA METHYLATION ANALYSIS

Pierangela Grignani[1], Barbara Bertoglio[1*], Maria Cristina Monti[1], Riccardo Cuoghi Costantini[2], Ugo Ricci[3], Martina Onofri[4], Paolo Fattorini[5], Carlo Previderè[1]

[1] Dipartimento di Sanità Pubblica, Medicina Sperimentale e Forense, Università di Pavia, Pavia, Italy

[2] Dipartimento di Scienze Biomediche, Metaboliche e Neuroscienze, Università di Modena e Reggio Emilia, Italy

[3] AOU Careggi SOD Diagnostica Genetica Equipe Genetica Forense, Firenze, Italy

[4] Dipartimento di Medicina e Chirurgia, Azienda Ospedaliera S. Maria, Università di Perugia, Terni, Italy

[5] Dipartimento Clinico di Scienze mediche, chirurgiche e della salute, Università di Trieste, Trieste, Italy

[*]Corresponding author: Barbara Bertoglio, Laboratorio di Genetica Forense, Dipartimento di Sanità Pubblica, Medicina Sperimentale e Forense, Università di Pavia, via Forlanini, 12, 27100 PAVIA, Italy, email: barbara.bertoglio@unipv.it

Fig S2 Graphical representation of the relationship between DNA methylation levels and chronological age and summary of the linear regression statistics for each CpG site. a. ELOVL2, b. FHL2, c. KLF14, d. C1orf132, e. TRIM59 (SE: standard error, 95% CI: 95% confidence interval, t: t-statistic, p: p-value)

a

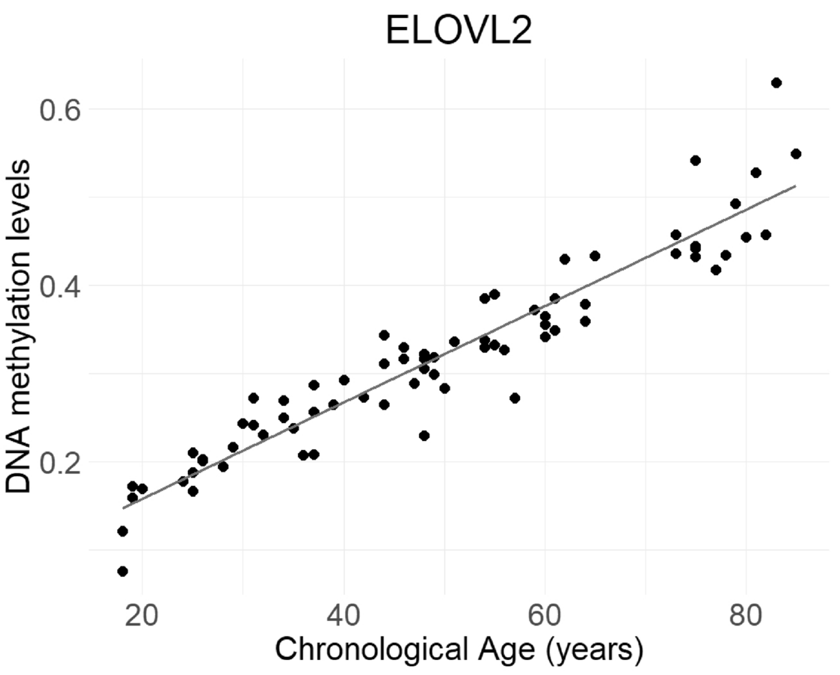

Parameter	Coefficient	SE	95% CI	t(70)	p
(Intercept)	-3.05	2.24	(-7.53, 1.42)	-1.36	0.178
ELOVL2	164.69	6.68	(151.37, 178.01)	24.66	< .001

Model: Age ~ ELOVL2 (72 Observations)
Residual standard deviation: 6.129 (df = 70)
R2: 0.897; adjusted R2: 0.895

b

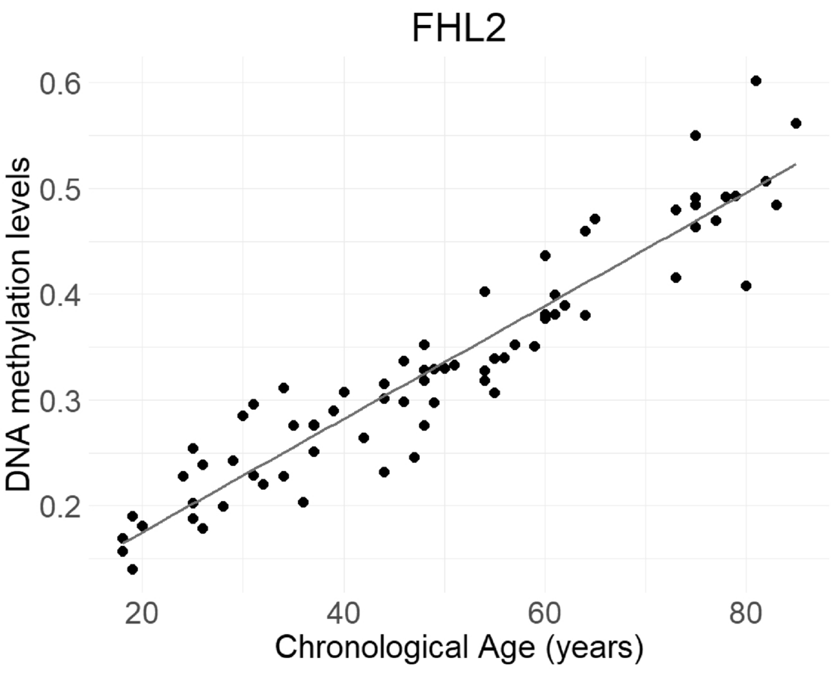

Parameter	Coefficient	SE	95% CI	t(70)	p
(Intercept)	-5.92	2.44	(-10.80, -1.05)	-2.42	0.018
FHL2	166.45	7.01	(152.47, 180.43)	23.74	< .001

Model: Age ~ FHL2 (72 Observations)
Residual standard deviation: 6.341 (df = 70)
R2: 0.890; adjusted R2: 0.888

c

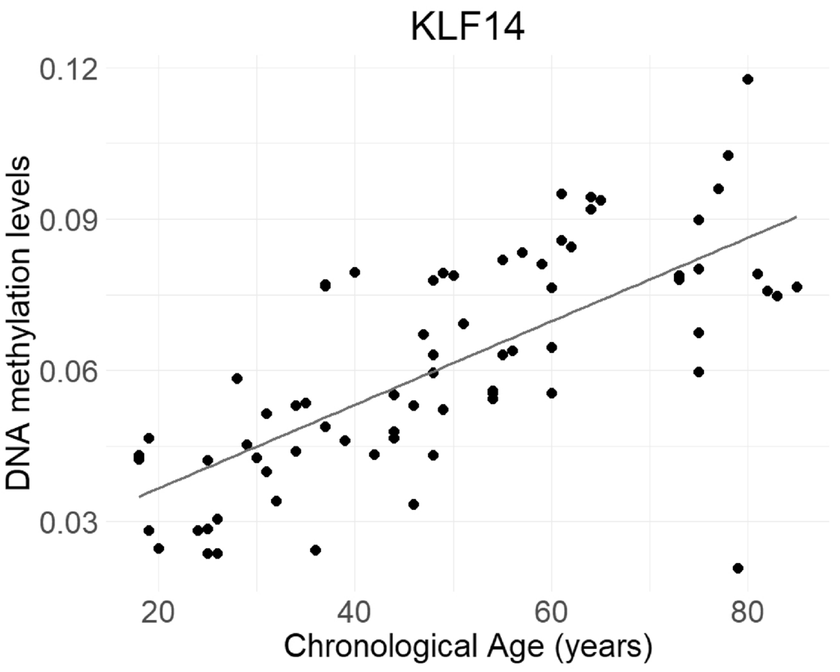

Parameter	Coefficient	SE	95% CI	t(70)	p
(Intercept)	12.62	4.68	(3.29, 21.95)	2.70	0.009
KLF14	602.45	72.20	(458.45, 746.44)	8.34	< .001

Model: Age ~ KLF14 (72 Observations)
Residual standard deviation: 13.509 (df = 70)
R2: 0.499; adjusted R2: 0.492

d

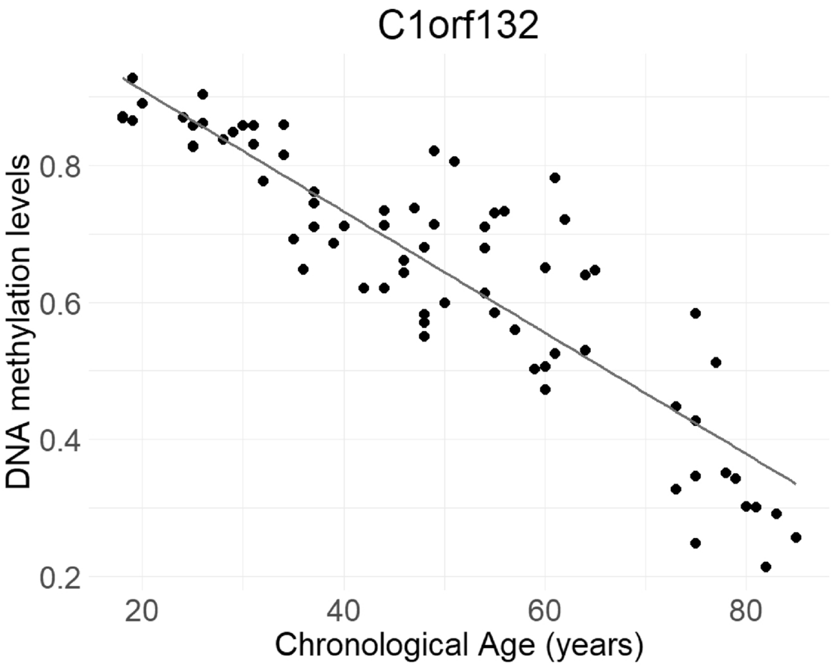

Parameter	Coefficient	SE	95% CI	t(70)	p
(Intercept)	108.21	3.66	(100.92, 115.51)	29.58	< .001
C1orf132	-90.57	5.41	(-101.36, -79.78)	-16.74	< .001

Model: Age ~ C1orf132 (72 Observations)
Residual standard deviation: 8.530 (df = 70)
R2: 0.800; adjusted R2: 0.797

e

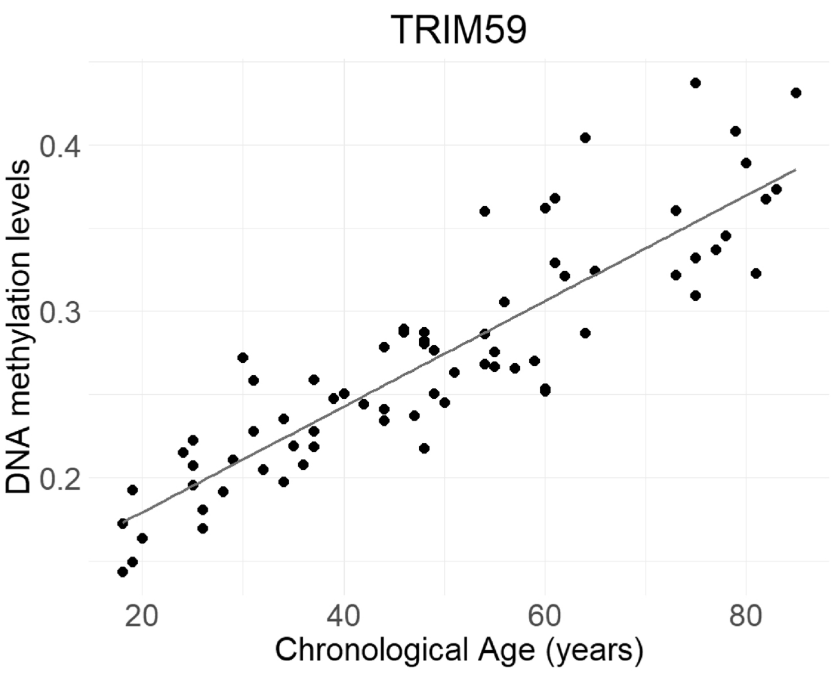

Parameter	Coefficient	SE	95% CI	t(70)	p
(Intercept)	-17.90	4.36	(-26.59, -9.22)	-4.11	< .001
TRIM59	246.88	15.53	(215.91, 277.86)	15.90	< .001

Model: Age ~ TRIM59 (72 Observations)
Residual standard deviation: 8.886 (df = 70)
R2: 0.783; adjusted R2: 0.780
