# Supplementary material for: Age estimation of burnt human remains through DNA methylation analysis
Source: Int J Legal Med. 2024 Sep 13;139(1):175–85. doi: 10.1007/s00414-024-03320-1 (PMC11732892; doi:10.1007/s00414-024-03320-1)
Supplement: Supplementary file 3 — Supplementary Material 3 [file 414_2024_3320_MOESM3_ESM.pdf]

AGE ESTIMATION OF BURNT HUMAN REMAINS THROUGH DNA METHYLATION ANALYSIS

Pierangela Grignani[1], Barbara Bertoglio[1*], Maria Cristina Monti[1], Riccardo Cuoghi Costantini[2], Ugo Ricci[3], Martina Onofri[4], Paolo Fattorini[5], Carlo Previderè[1]

[1] Dipartimento di Sanità Pubblica, Medicina Sperimentale e Forense, Università di Pavia, Pavia, Italy

[2] Dipartimento di Scienze Biomediche, Metaboliche e Neuroscienze, Università di Modena e Reggio Emilia, Italy

[3] AOU Careggi SOD Diagnostica Genetica Equipe Genetica Forense, Firenze, Italy

[4] Dipartimento di Medicina e Chirurgia, Azienda Ospedaliera S. Maria, Università di Perugia, Terni, Italy

[5] Dipartimento Clinico di Scienze mediche, chirurgiche e della salute, Università di Trieste, Trieste, Italy

[*]Corresponding author: Barbara Bertoglio, Laboratorio di Genetica Forense, Dipartimento di Sanità Pubblica, Medicina Sperimentale e Forense, Università di Pavia, via Forlanini, 12, 27100 PAVIA, Italy, email: barbara.bertoglio@unipv.it

| Parameter | Coefficient | SE | 95% CI | t(66) | p |
|---|---|---|---|---|---|
| (Intercept) | 17.66 | 5.58 | (6.52, 28.79) | 3.17 | 0.002 |
| ELOVL2 | 61.66 | 11.81 | (38.07, 85.25) | 5.22 | < .001 |
| FHL2 | 35.05 | 12.58 | (9.94, 60.17) | 2.79 | 0.007 |
| KLF14 | 136.28 | 26.01 | (84.36, 188.21) | 5.24 | < .001 |
| C1orf132 | -25.61 | 4.57 | (-34.73, -16.49) | -5.61 | < .001 |
| TRIM59 | 32.22 | 14.14 | (3.99, 60.45) | 2.28 | 0.026 |

Model: Age ~ ELOVL2 + FHL2 + KLF14 + C1orf132 + TRIM59 (72 Observations)
Residual standard deviation: 3.708 (df = 66)
$R^2$: 0.964; adjusted $R^2$: 0.962
F-statistic: 357.4 on 5 and 66 df, p-value: 2.30e-46

**Table S1** Summary statistics of the multivariate linear regression model of the blood samples from living individuals (SE: standard error, 95% CI: 95% confidence interval, t: t-statistic, p: p-value)

| **Parameter** | **Coefficient** | **SE** | **95% CI** | **t(65)** | **p** |
| --- | --- | --- | --- | --- | --- |
| (Intercept) | 17.94 | 5.58 | (6.79, 29.09) | 3.21 | 0.002 |
| ELOVL2 | 64.22 | 12.07 | (40.11, 88.33) | 5.32 | < .001 |
| FHL2 | 30.32 | 13.40 | (3.55, 57.09) | 2.26 | 0.027 |
| KLF14 | 141.66 | 26.53 | (88.68, 194.63) | 5.34 | < .001 |
| C1orf132 | -25.50 | 4.57 | (-34.62, -16.38) | -5.58 | < .001 |
| TRIM59 | 34.16 | 14.26 | (5.68, 62.65) | 2.40 | 0.019 |
| Sex (M) | -0.96 | 0.94 | (-2.85, 0.92) | -1.02 | 0.311 |

Model: Age ~ ELOVL2 + FHL2 + KLF14 + C1orf132 + TRIM59 + Sex (72 Observations)
Residual standard deviation: 3.707 (df = 65)
$R^2$: 0.965; adjusted $R^2$: 0.962
F-statistic: 298.2 on 6 and 65 df, p-value: 2.74e-45

**Table S2** Summary statistics of the multivariate linear regression model of the blood samples from living individuals with sex as further independent variable (SE: standard error, 95% CI: 95% confidence interval, t: t-statistic, p: p-value)

| | **Adj. $R^2$** | **MAE** | **RMSE** | **AIC** | **BIC** | **p-value** |
|---|---|---|---|---|---|---|
| **Model** | 0.962 | 2.84 | 3.55 | 400.79 | 416.73 | <0.001 |
| **Model with sex** | 0.962 | 2.82 | 3.52 | 401.65 | 419.86 | <0.001 |

**Table S3** Summary statistics of the multivariate linear regression models. Comparison between the models with and without sex (Adj. $R^2$: adjusted $R^2$, MAE: Mean Absolute Error, RMSE: Root Mean Square Error, AIC: Akaike Information Criterion, BIC: Bayesian Information Criterion, p-value: F-test p-value)

| | $R^2$ | MAE | RMSE |
|---|---|---|---|
| **Model** | 0.962 | 2.84 | 3.55 |
| **Validation** | 0.962 | 3.15 | 3.88 |

**Table S4** Summary of the accuracy parameters for the model and model validation (MAE: Mean Absolute Error, RMSE: Root Mean Square Error)

**Correctness of prediction**

| Variable | Category | n | OR | 95% CI | | Wald test p-value | Anova test p-value |
|---|---|---|---|---|---|---|---|
| Glassman score | | | 0.63 | 0.27 | 1.46 | 0.280 | 0.262 |
| Pope score | | | 0.88 | 0.48 | 1.59 | 0.666 | 0.662 |
| Environment | V | 20 | Reference | | | | 0.354 |
| Environment | R | 8 | 3.40 | 0.52 | 22.41 | 0.203 | 0.354 |
| Environment | O | 1 | NC | NC | NC | NC | 0.354 |
| TDC | | | 1.00 | 0.95 | 1.05 | 0.977 | 0.977 |
| **DI** | | | **3.09** | **1.21** | **7.85** | **0.018** | **0.004** |
| **DI >2** | **No** | **22** | **Reference** | | | | **0.010** |
| **DI >2** | **Yes** | **7** | **13.33** | **1.65** | **107.43** | **0.015** | **0.010** |

**Prediction error**

| Variable | Category | n | MD | 95% CI | | Wald test p-value | Anova test p-value |
|---|---|---|---|---|---|---|---|
| Glassman score | | | 0.27 | -2.48 | 3.02 | 0.848 | 0.847 |
| Pope score | | | 0.84 | -1.17 | 2.85 | 0.420 | 0.413 |
| Environment | V | 20 | Reference | | | | 0.745 |
| Environment | R | 8 | 1.44 | -5.81 | 8.68 | 0.701 | 0.745 |
| Environment | O | 1 | -5.55 | -23.31 | 12.20 | 0.545 | 0.745 |
| TDC | | | 0.00 | -0.19 | 0.19 | 0.978 | 0.978 |
| **DI** | | | **2.54** | **0.14** | **4.93** | **0.048** | **0.038** |
| **DI >2** | **No** | **22** | **Reference** | | | | **0.049** |
| **DI >2** | **Yes** | **7** | **7.01** | **0.04** | **13.99** | **0.059** | **0.049** |

**DI**

| Variable | Category | n | MD | 95% CI | | Wald test p-value | Anova test p-value |
|---|---|---|---|---|---|---|---|
| Glassman score | | | -0.07 | -0.47 | 0.34 | 0.749 | 0.747 |
| Pope score | | | -0.09 | -0.39 | 0.20 | 0.535 | 0.529 |
| Environment | V | 20 | Reference | | | | 0.771 |
| Environment | R | 8 | 0.35 | -0.71 | 1.41 | 0.527 | 0.771 |
| Environment | O | 1 | -0.33 | -2.93 | 2.27 | 0.804 | 0.771 |
| TDC | | | -0.01 | -0.03 | 0.02 | 0.698 | 0.695 |

**Table S5** Association analyses between the correctness of prediction/prediction error and sample variables, i.e., thermal damage scores (i.e., Glassman score and Pope score), fire environments (Environment V = vehicle fire, Environment R = residential structure fire, Environment O = outdoor space fire), time from death to sample collection (TDC), and DNA degradation index (DI). Mean deviation (MD)/Odds Ratio (OR) are reported for each variable with 95% confidence interval (95% CI), anova statistics and Wald test p-values (n = number of samples). Association analyses are performed also between DI and thermal damage scores, fire environments, and TDC. Significant associations are highlighted in bold.

Only the degradation index (DI) showed a significant association. In order to identify the most discriminative DI values with respect to the predictive accuracy of the model, a classification and regression tree (CART) analysis was applied suggesting a split cut-off of 2. Classifying the sample set into two categories (i.e., DI>2 yes/no), the significant association with the correctness of prediction and prediction error was confirmed
